# Evaluation of Two Methods for the Detection of Third Generation Cephalosporins Resistant Enterobacterales Directly From Positive Blood Cultures

**DOI:** 10.3389/fcimb.2020.00491

**Published:** 2020-09-11

**Authors:** Clarisse Durand, Agathe Boudet, Jean-Philippe Lavigne, Alix Pantel

**Affiliations:** ^1^Service de Microbiologie et Hygiène Hospitalière, CHU Nîmes, Nîmes, France; ^2^VBMI, INSERM U1047, Université de Montpellier, Service de Microbiologie et Hygiène Hospitalière, CHU Nîmes, Nîmes, France

**Keywords:** bacteremia, BL-RED^TM^ test, β-LACTA^TM^ test, blood cultures, extended-spectrum beta-lactamases, multidrug resistance, third-generation cephalosporins

## Abstract

Due to the importance of a rapid determination of patients infected by multidrug resistant bacteria, we evaluated two rapid diagnostic tests for the detection of third-generation cephalosporins (3GC)-resistant Enterobacterales directly from positive blood cultures within 1 h: BL-RED^TM^ (electrochemical method) and β-LACTA^TM^ test (chromogenic method). A panel of 150 clinical strains characterized for their resistance profiles (e.g., penicillinases, extended-spectrum beta-lactamases (ESBLs), overproduction of cephalosporinase, carbapenemases, impermeability) was tested. Approximately 100 CFU of each isolate was spiked into sterile blood culture bottles and incubated in a BD BACTEC^TM^ FX automated system (Becton Dickinson, USA). Positive blood cultures were examined to parallel testing using the BL-RED^TM^ and β-LACTA^TM^ tests and conventional susceptibility method (disc diffusion following EUCAST recommendations). For all phenotypes combined, the sensitivity, specificity, positive predictive value, and negative predictive value in the detection of 3GC resistance were, respectively (i) with BL-RED^TM^: 45.7, 100, 100, and 54.2% and (ii) with β-LACTA^TM^ test: 52.2, 100, 100, and 56.9%. The positivity of tests allows to adapt antibiotic treatment whereas the negative result requires other tests. Moreover, these tests detect most Ambler class A-producing Enterobacterales (KPC, ESBL, extended-spectrum OXY) with sensitivities and specificities of 87.5 and 99% for BL-RED^TM^, respectively and both 100% for β-LACTA^TM^ test (47/47 isolates). These two rapid tests failed to detect AmpC overexpressed (sensitivities of 2.7% for BL-RED^TM^ and 0% for β-LACTA^TM^ test) and Ambler class B-producing Enterobacterales (sensitivities of 40% for both tests) notably strains without ESBLs associated (sensitivities of 0% for both tests). BL-RED^TM^ and β-LACTA^TM^ tests are easy-to-use and mainly attractive when a positive result is obtained notably to detect most of the Ambler class A-producing Enterobacterales in <1 h after the positivity of the blood culture, allowing a rapid adaptation of the antibiotic therapy in patients.

## Introduction

The spread of multidrug resistant (MDR) Enterobacterales is a major public health problem notably related to the misuse and overuse of antibiotics (Laxminarayan et al., 2013; World Health Organisation, 2014). Extended-spectrum cephalosporins and carbapenems are currently become the first line to treat community-acquired and nosocomial infections (Coque et al., 2008), involving a growing problem of the expansion of non-susceptibility of Enterobacterales to third-generation cephalosporins (3GC) and carbapenems. This increase is largely due to the importance of extended-spectrum β-lactamases (ESBL), but also to the overproduced chromosomal or plasmid-mediated AmpC cephalosporinases and to the emergence of carbapenemases (Peirano and Pitout, 2019). The rapid optimization of antibiotic therapy, according to the organism and its resistance profile, is a major goal both for individual patients and for public health (Roca et al., 2015). Bloodstream infections (BSI) are 30% of admissions in ICU and is responsible for an increase in length of stay and a leading cause of mortality (close to 40%) (Kang et al., 2005). The importance of the early appropriate treatment is crucial in these pathologies, given the linear increase in the risk of mortality with each hour for which administration is delayed (Ibrahim et al., 2000; Ferrer et al., 2014). However, conventional microbiological methods are time consuming requiring 12–48 h to identify the causal microorganism and provide an antimicrobial susceptibility testing (AST). Over the last couple of years, the improvement of available rapid diagnostic tests directly from positive blood cultures has changed approaches for identification and AST (Maugeri et al., 2019). These tests have the advantage of providing fast results, optimizing antibiotic therapy, improving survival, and reducing the length of hospitalization (Perez et al., 2013). These new techniques promote the proper use of antibiotics by limiting the antimicrobial resistance and decreasing the medico-economic impact by (de)-escalation of the antibiotic therapy and reduction of hospital stay (Farfour et al., 2019). They include molecular biology tests identifying the MDR encoding genes (pathogen-specific real-time or multiplex PCR), spectrometry assays detecting β-lactamase hydrolytic activity (MALDI TOF-MS), biochemical tests, β-lactamases inhibitor-based tests, chromogenic tests and electrochemical test (Buchan et al., 2013; Renvoise et al., 2013; Bogaerts et al., 2016; Salimnia et al., 2016; Faron et al., 2017; Pantel et al., 2018).

The aim of the present study was to evaluate the performance of: (i) a new electrochemical test, BL-RED^TM^ (Beta-Lactamase Rapid Electrochemical Detection, CORIS BioConcept, Belgium); and (ii) a colorimetric test, β-LACTA^TM^ test (Bio-Rad, Marnes la Coquette, France), for the detection of 3GC-resistance on a panel of Enterobacterales directly from positive blood cultures within 1 h.

## Materials and Methods

### Bacterial Panel

A panel of 150 Enterobacterales isolates from our regional MDR Gram-negative Bacilli Reference Lab (CARB-LR group) in the Occitanie region was tested (Pantel et al., 2014; Robert et al., 2014). The isolates were included with the following distribution: *Escherichia coli* (*n* = 62), *Klebsiella pneumoniae* (*n* = 29), *Enterobacter cloacae* (*n* = 20), *Klebsiella aerogenes* (*n* = 9), *Proteus mirabilis* (*n* = 7), *Morganella morganii* (*n* = 6), *Klebsiella oxytoca* (*n* = 5), *Citrobacter freundii* (*n* = 5), *Citrobacter koseri* (*n* = 2), *Providencia rettgeri* (*n* = 2), *Serratia marcescens* (*n* = 2), and *Hafnia alvei* (*n* = 1).

Different β-lactam resistance profiles were selected: (i) 3GC-susceptible isolates (*n* = 50), (ii) ESBL producers (*n* = 41, including three which also overproduced AmpC), (iii) chromosomal-hyperproduced (*n* = 36) or plasmid-mediated (*n* = 10) AmpC producers, (iv) extended-spectrum OXY (*n* = 3), and (v) carbapenemase producers (*n* = 30, including 12 ESBL, 4 chromosomal AmpC, and 1 plasmid-mediated AmpC). Twenty-two isolates producing ESBL, high level AmpC or OXY were resistant to ertapenem by membrane permeability alteration. The presence of ESBLs, plasmid mediated AmpC, and carbapenemases genes was previously confirmed by specific PCR and sequence analysis (Perez-Perez and Hanson, 2002; Pitout et al., 2004, 2007; Poirel et al., 2011). All characteristics of the strains are summarized in Table 1.

**Table 1:** Characteristics of the studied Enterobacterales isolates.

**Resistance profile (no. of strains)**		**Species (no. of strains)**	**β-lactamase content (no. of strains)**
3GC susceptible (50)	No resistance to β-lactams (19)	*E. coli* (18)	None (18)
		*P. mirabilis* (1)	None (1)
	Penicillinases (22)	*E. coli* (17)	TEM-1 (10)
			Inhibitor-resistant TEM (7)
		*K. pneumoniae* (3)	SHV-1 (2)
			Inhibitor-resistant TEM (1)
		*C. koseri* (2)	CKO (2)
	Cephalosporinases (9)	*M. morganii* (3)	Low-level AmpC (3)
		*P. rettgeri* (2)	Low-level AmpC (2)
		*K. aerogenes* (1)	Low-level AmpC (1)
		*E. cloacae* (1)	Low-level AmpC (1)
		*E. coli* (1)	Low-level AmpC (1)
		*S. marcescens* (1)	Low-level AmpC (1)
β-lactamases conferring resistance to 3GC (70)	ESBL (29)	*E. coli* (16)	CTX-M-group 1 (7)
			CTX-M-group 1 + CTX-M-group 8 (5)
			CTX-M-group 9 (4)
		*P. mirabilis* (5)	CTX-M-group 1 (3)
			CTX-M-group 9 (1)
			CTX-M no group typed (1)
		*K. pneumoniae* (4)	CTX-M-group 1 + SHV-1 (2)
			SHV-5 (1)
			CTX-M-group 1 + CTX-M-group 8 + SHV-1 (1)
		*E. cloacae* (3)	CTX-M-group 1 + High-level AmpC (1)
			CTX-M-group 9 + High-level AmpC (1)
			TEM-24 + High-level AmpC (1)
		*K. oxytoca* (1)	CTX-M-group 8 + OXY-1 (1)
	Chromosomal overproduced cephalosporinases (29)	*E. cloacae* (9)	High-level AmpC (9)
		*E. coli* (6)	High-level AmpC (6)
		*K. aerogenes* (6)	High-level AmpC (6)
		*C. freundii* (4)	High-level AmpC (4)
		*M. morganii* (3)	High-level AmpC (3)
		*H. alvei* (1)	High-level AmpC (1)
	Plasmid cephalosporinases (9)	*K. pneumoniae* (9)	DHA-1 + SHV-1 (9)
	Chromosomal overproduced penicillinases (3)	*K. oxytoca* (3)	High-level OXY-1 (3)
Carbapenemases (30)	Class A carbapenemase (6)	*K. pneumoniae* (5)	KPC-2 + SHV-1 (4)
			KPC-2 + CTX-M-group 1 + SHV-1 (1)
		*E. cloacae* (1)	IMI-1 + Low-level AmpC (1)
	Class B carbapenemase (10)	*E. cloacae* (4)	VIM-1 + High-level AmpC (3)
			VIM-1 + CTX-M-group 9 (1)
		*E. coli* (2)	NDM-1 + CTX-M-group 9 (1)
			NDM-1 + DHA (1)
		*K. pneumoniae* (2)	NDM-1 + CTX-M-group 1 + SHV-1 (2)
		*C. freundii* (1)	VIM-1 + High-level AmpC (1)
		*P. mirabilis* (1)	NDM-1 (1)
	Class D carbapenemase (12)	*K. pneumoniae* (4)	OXA-48 + SHV-1 (2)
			OXA-48 + CTX-M-group 1 + SHV-1 (2)
		*K. aerogenes* (2)	OXA-48 + Low-level AmpC (2)
		*E. cloacae* (2)	OXA-48 + CTX-M-group 1 + Low-level AmpC (2)
		*E. coli* (2)	OXA-48 (1)
			OXA-181 + CTX-M-group 1 (1)
		*K. oxytoca* (1)	OXA-48 + OXY-1 (1)
		*S. marcescens* (1)	OXA-48 + Low-level AmpC (1)
	Class B + D carbapenemase (2)	*K. pneumoniae* (2)	NDM-1 + OXA-48 + CTX-M-group 1 + SHV-1 (2)

### Sample Preparation

For each isolate, ~100 CFU was spiked into sterile blood cultures [BD BACTEC^TM^ Plus Aerobic/F and BD BACTEC^TM^ Lytic/10 Anaerobic/F (BD Diagnostics, Le Pont de Claix, France)] containing 10 mL of fresh blood. Blood cultures were incubated in a BACTEC^TM^ FX automated blood culture device until they flagged positive for microbial growth. All positive blood cultures were divided into three samples: 1 mL for the conventional comparator methods, 1 mL for the β-LACTA^TM^ test (Bio-Rad, Marnes-la-Coquette, France) and 0.5 mL for the BL-RED^TM^ test (Coris BioConcept, Gembloux, Belgium).

### Conventional Comparator Method

The positive blood cultures were subcultured on blood agar (bioMérieux, Marcy l'Etoile, France) for 18–24 h at 37 ± 2°C. Species-level identification was performed on isolated colonies on blood agar by the mass spectrometry (Vitek® MS, bioMérieux). AST was determined by the agar disc-diffusion method according to the EUCAST-SFM 2019 recommendations (www.eucast.org). The results of disc diffusion were used as the comparator for the 3GC resistance of each isolate (determined by the agar disc-diffusion size), using notably: ceftazidime 10 μg and cefotaxime 5 μg discs.

### BL-RED^TM^ Test V1.0

All blood cultures were tested after the blood culture flagged positive as recommended by the manufacturer. For aerobic blood culture, a 40 μL sample was mixed with 40 μL of reagent and incubated 1 h at 37 ± 2°C. For anaerobic blood culture, a pre-analysis was needed: a 500 μL sample was centrifuged one time 2 min at 6,000 g; the pellet was re-suspended into 500 μL of NaCl solution (0.9%) and re-centrifuged 2 min at 6,000 g; this second pellet was suspended into 40 μL of buffer and 40 μL of reagent; the mix was incubated 1 h at 37 ± 2°C. After this incubation, 20 μL of the mix was placed on the ceramic electrode and after in the sensor. The reagent includes a “false” 3GC substrate which is hydrolysed when there is a 3GC β-lactamase, this frees an electro-conductive product. The intensity of the electrochemical signal [measured by DropSens (Metrohm, Villebon Courtaboeuf, France)] is posted in nanoAmpere (nA). The threshold of positivity is 80 nA for blood culture.

### β-LACTA^TM^ Test

The protocol B of the manufacturer recommendations was followed for the treatment of the positive blood cultures. Three different centrifugations and different solutions (Triton 0.01%, NaCl solution) were used to obtain the final bacterial pellet. After using the reagents provided in the kit on the bacterial pellet, we can read visually the hydrolysis of the chromogenic substrate (HMRZ-86, cephalosporin) from yellow to red in 15 min for a positive test. No change in color was considered a negative result (no hydrolysis of HMRZ-86).

### Statistical Analysis

The sensitivity, specificity, negative and positive predictive value (NPV and PPV, respectively) were calculated and comparison with molecular characterization which served as the reference standard. 3GC-sensitive isolate with a negative test and 3GC-resistant isolate with a positive test are considered correct. Conversely, 3GC-sensitive isolate with a positive test and 3GC-resistant isolate with a negative test are considered incorrect. Additionally, the 95% confidence intervals (CIs), accuracy and the Youden index were calculated.

## Results

### Evaluation on 3GC-Susceptible Isolates

Among our panel, 50 isolates had no 3GC resistance. These isolates harbored either a penicillinase (CKO, TEM-1, SHV-1, inhibitor-resistant TEM) or a naturally chromosomally-encoded inducible AmpC cephalosporinase. The two assays confirmed the absence of 3GC-resistance in all strains: 0 nA for most of the strains with BL-RED^TM^, yellow color with β-LACTA^TM^ test.

Of note, two isolates (one wild-type *E. coli* and one TEM-1-producing *E. coli*) presented a very low intensity using BL-RED^TM^ at 10 nA. However, they were still considered negative because the positive threshold for blood culture was established at 80 nA.

### Global Detection of 3GC-Resistance

Concerning all isolates belonging to the tested panel, the performance of the BL-RED^TM^ and β-LACTA^TM^ tests were, respectively: sensitivity, 46.7% (CI 36.9–56.9%) and 52.2% (CI 42.1–62.1%); specificity, both 100% (CI 93.8–100%); NPV, 54.2% (CI 44.8–63.3%), and 56.9% (CI 47.2–66.1%) and PPV, both 100% (CI 91.8–100%; 92.6–100%). The Youden index was 46.7 and 52.2%, respectively (Table 2).

**Table 2:** Analytical performance of assays for detection of 3GC-resistant Enterobacterales isolates in positive blood cultures.

**Method**	**Result**	**3GC cephalosporins**		**Sensitivity (95% CI)**	**Specificity (95% CI)**	**PPV (95% CI)**	**NPV (95% CI)**	**Accuracy (95% CI)**	**Youden**
		**Resistant (*n* = 92)**	**Susceptible (*n* = 58)**						
BL-RED^TM^	Positive	43	0	46.7% (36.9–56.9)	100% (93.8–100)	100% (91.8–10)	54.2% (44.8–63.3)	67.3%	46.7%
	Negative	49	58						
β-LACTA^TM^	Positive	48	0	52.2% (42.1–62.1)	100% (93.8–100)	100% (92.6–100)	56.9% (47.2–66.1)	70.7%	52.2%
	Negative	44	58						

*PPV, positive predictive value; NPV, negative predictive value*.

### Evaluation on Ambler Class a β-Lactamase Producers

When considering only the Ambler class A ESBL (without carbapenemase associated), 79.3% (23/29) of the isolates were positive using the BL-RED^TM^, with a signal >80 nA [100–1,220] (Table S1). Six isolates were negative: two CTX-M-group 1-producing *P. mirabilis*, one CTX-M-group 9-producing *E. coli*, one CTX-M-group 1 and 8-producing *E. coli*, one CTX-M-group 9-producing *E. cloacae* with altered membrane permeability, and one SHV-5-producing *K. pneumoniae* with altered membrane permeability. All these isolates (*n* = 29) were positive using the β-LACTA^TM^ test that shows a sensitivity of ESBL detection of 100%. Interestingly, the two techniques detected the OXY-hyperproducing *K. oxytoca* isolates with low outer membrane permeability (*n* = 3).

Concerning Ambler class A carbapenemases, both tests were able to detect the KPC-producing isolates (*n* = 5) whatever the presence or absence of an ESBL (Table S1). Negative results were obtained with IMI-1-producing *E. cloacae* strain that is resistant to carbapenems but remains susceptible to 3GC.

The analysis of the different previous results suggested that the two methods evaluated in this study had an interest in the detection of Ambler class A producers (not restricted to ESBL producers). When considering all Ambler class A producers (excluding IMI producers), the performance of BL-RED^TM^ and β-LACTA^TM^ were, respectively: sensitivity, 87.5% (CI 75.3–94.1%) and 100% (CI 92.6–100%); specificity, 99.0% (CI 94.7–99.8%) and 100% (CI 96.4–100%); PPV, 97.7% (CI 87.9–99.6%) and 100% (CI 92.6–100%) and NPV, 94.4% (CI 88.3–97.4%) and 100% (CI 96.4–100%) (Table 3).

**Table 3:** Analytical performance of assays for detection of Ambler Class A β-lactamases-producing Enterobacterales isolates in positive blood cultures.

**Method**	**Result**	Class A b-lactamase^*^		**Sensitivity (95% CI)**	**Specificity (95% CI)**	**PPV (95% CI)**	**NPV (95% CI)**	**Accuracy (95% CI)**	**Youden**
		**Resistant (*n* = 37)**	**Susceptible (*n* = 113)**						
BL-RED^TM^	Positive	31	1	83.8% (68.9–92.4)	99.1% (95.2–99.8)	96.9% (84.3–99.5)	94.9% (89.4–97.7)	95.3%	82.9%
	Negative	6	112						
β-LACTA^TM^	Positive	37	0	100% (90.6–100)	100% (96.7–100)	100% (90.6–100)	100% (96.7–100)	100%	100%
	Negative	0	113						

* *IMI-producing E. cloacae strain susceptible to 3GC was excluded*. *PPV, Positive Predictive Value; NPV, Negative Predictive Value*.

### Evaluation on Ambler Class C β-Lactamase Producers

All AmpC-overproducing isolates (*n* = 29, excluding ESBL co-producers) tested were negative for the two techniques (Table S2). The alteration of outer membrane permeability (seven strains) did not influence the results. One isolate (*C. freundii*) presented a high intensity using BL-RED^TM^ at 60 nA but under the positive threshold. The antibiogram analysis performed after blood cultures confirmed the resistance to 3GC and the presence of the hyperproduction of AmpC in all isolates.

Among plasmid-mediated AmpC producers (*n* = 10), only one strain (*K. pneumoniae* DHA-1 with decreased membrane permeability) was positive with BL-RED^TM^ test (480 nA).

### Evaluation on Ambler Class B and D β-Lactamase Producers

Despite the high level of 3GC resistance, Ambler class B carbapenemase producers were only detected (6/12) in both tests when the isolates were also ESBL co-producers.

Similarly, OXA-48 carbapenemases were only detected (7/14) by the two tests when the isolates were ESBL co-producers.

## Discussion

Enterobacterales are the most important etiologies of community-onset and hospital-acquired BSI (Laupland and Church, 2014). The diffusion of 3GC-resistance among Enterobacterales, resulting from the spread of ESBL- and carbapenemase-producing isolates represents a serious problem worldwide. This is particularly true for life-threatening infections such as BSI, for which inappropriate first-line antibiotic therapy has a dramatic impact on short-term mortality (Kumar et al., 2009; Zilberberg et al., 2014).

Rapid and reliable identification of MDR Enterobacterales in the clinical microbiology laboratory is essential to effective infection control. In this study, we evaluated two rapid and easy-to-use methods: BL-RED^TM^ and β-LACTA^TM^ tests. If the β-LACTA^TM^ test has been used for few years on bacterial strains and directly from blood cultures, the present study is the first to evaluate the recently commercialized BL-RED^TM^ test v1.0. Both tests allowed early detection of most Ambler class A β-lactamases conferring 3GC resistance (e.g., ESBL, OXY, and KPC) to Enterobacterales (Table 3) in <1 h after positivity of the blood culture with interesting performance (sensitivity and specificity to 83.8 and 99.1% for BL-RED^TM^ test and both 100% for β-LACTA^TM^ test) suggesting that the two tests may provide useful therapeutic guidance. However, when testing challenged 3GC-resistant isolates non-ESBL producers, the two tests showed poor performance. Of note, a very low detection rate of plasmid-mediated AmpC or AmpC-overproducing isolates could be observed: 2.4% for BL-RED^TM^ and 0% for β-LACTA^TM^ test. This limitation has been previously published concerning β-LACTA^TM^ test, when testing with subculture on agar plates (Renvoise et al., 2013; Morosini et al., 2014; Compain et al., 2015). The lack of sensitivity for the detection of high level AmpC must be balanced against the fact that ESBL producers are predominant among 3GC resistant Enterobacterales in European countries, notably among BSI (European Antimicrobial Resistance Surveillance Network (EARS-Net), 2018). Moreover, early adaptation by cefepime could be proposed when naturally AmpC producer was identified by MALDI-TOF MS, as previously suggested (Mizrahi et al., 2018). Another limitation of both tests was the failure to identify metallo-β-lactamase-producing isolates directly in blood cultures, whereas β-LACTA^TM^ test carried out with VIM-1-producers on agar plates showed acceptable sensitivity (Morosini et al., 2014). The lack of detection of metallo-β-lactamase-producing Enterobacterales isolates have been previously reported with phenotypical tests applied on blood culture, the zinc concentration in the medium being essential for an efficient detection of Ambler class B enzymes (Dortet et al., 2014; Pantel et al., 2018). Concerning class D carbapenemase producers, the isolates are frequently associated with ESBL positive producers (Bakthavatchalam et al., 2016) allowing their detection with both evaluated tests (7/7, 100%). Without the presence of ESBL-carrying plasmids, the tests failed to identify this resistance mechanism. To date, molecular methods remain the faster tool with a higher sensitivity to detect the carbapenemase-producing Enterobacterales (Rood and Li, 2017).

If no study has previously evaluated the BL-RED^TM^ test, some previous works were performed on the use of β-LACTA^TM^ test combined with MALDI-TOF MS from positive blood cultures (Compain et al., 2015; Walewski et al., 2015; Hasso et al., 2017; Mizrahi et al., 2018). The first study conducted in 2015 by Compain et al. evaluated the β-LACTA^TM^ test on 3-h subcultures. 84.8% of the 33 blood culture isolates resistant to 3GC were correctly detected Compain et al. (2015). Walewski et al. (2015) used the test on bacterial pellets from the blood culture broths after treatment by saponin followed by two washes, with 95.7% of sensitivity and 100% of specificity for identifying ESBL-producing Enterobacterales. Hasso et al. (2017) evaluated the accuracy of the β-LACTA^TM^ test for rapid detection of ESBL-producing *E. coli* and *Klebsiella* spp. from smudge plates prepared with positive blood cultures. The authors noted a sensitivity and specificity of 100 and 97.8%, respectively. Finally, the last two studies carried out in France by Mizrahi et al. (2018) and Farfour et al. (2019) obtained similar results: excellent performance regarding ESBL-producers and lack of sensitivity for Group 3 Enterobacterales. All results underlined the interest of these rapid techniques to be used effectively in daily laboratory practice.

**Figure 1:**
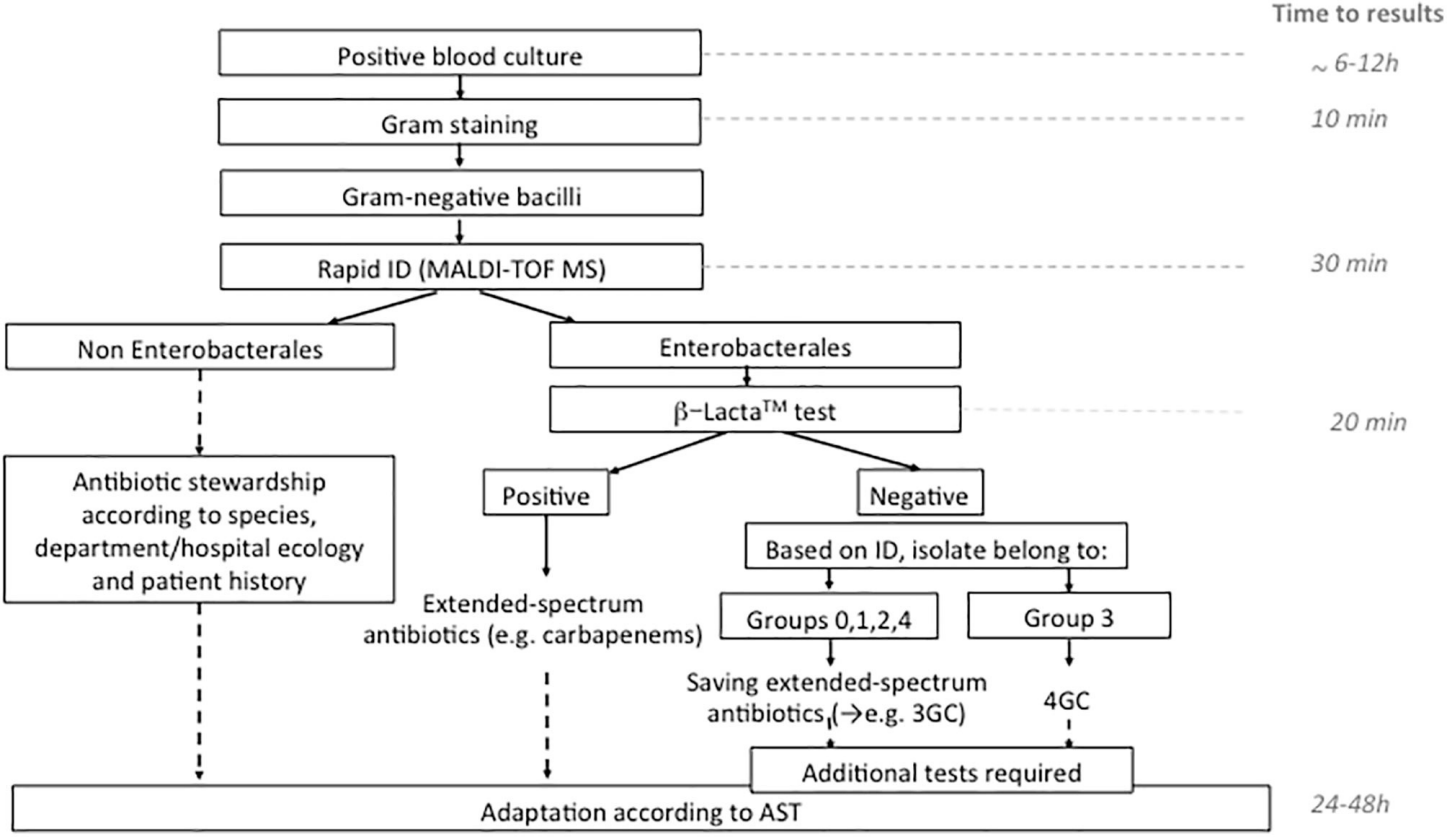
Algorithm proposed for the management of Gram negative bacilli in positive blood culture in the routine laboratory. ID, identification; 3GC, third generation cephalosporins; 4GC, fourth generation cephalosporin; AST, antimicrobial susceptibility testing.

The use of rapid phenotypic susceptibility techniques have demonstrated their interest in an optimized antimicrobial treatment (Schneider et al., 2019). Globally, our study shows excellent PPV (both 100%) but poor NPV (46.7 and 52.2%) for the BL-RED^TM^ and β-LACTA^TM^ tests, respectively. This reinforces the idea to only manage positive results with these assays and adapt antibiotic treatment whereas the negative results need to use additional tests. In this way, we suggest an algorithm incorporating MALDI-TOF MS combined with β-LACTA^TM^ test for optimized Ambler class A producers detection with the advantage of being rapid, affordable, and simple (Figure 1). This algorithm could be accurate to give in <2 h a decisive orientation in the antibiotic management of sepsis or septic shock patients, particularly when the β-LACTA^TM^ test is positive. An early adaptation by 4GC could be proposed when naturally AmpC producer was identified by MALDI-TOF MS with a negative β-LACTA^TM^ test. However, its use must be debated. Recently, Dépret et al. (2018) evaluated the rapid identification using MALDI-TOF MS results only vs. the combined MALDI-TOF MS and the β-LACTA^TM^ test in a method close to our algorithm. No impact on the current standard choice of therapy in terms of escalation/de-escalation or in reduction of carbapenems prescription was observed. Indeed, only 9% of the positive blood cultures in this study were confirmed as ESBL-producing, decreasing the interest of the techniques. This suggests that the use of a rapid test for Ambler class A-producers detection would be useful in a population with high prevalence of these enzymes and/or when treatment choices are not made by infectious disease specialists. In contrast, Mizrahi et al. (2018) have demonstrated the crucial role played by rapid detection of 3GC resistance with β-LACTA^TM^. In this study, 75% (21/28) of the patients with BSI involving 3GC-resistant bacteria received a non-adapted first-line treatment. β-LACTA^TM^ test performed on blood culture significantly reduced the delay for treatment adaptation (28.1 h) and patient isolation (35 h). In 2019, Farfour et al. (2019) observed the same impact of rapid diagnostic tests on the management of BSI. Patients receiving a rapid strategy (rapid identification and 3GC-susceptibility testing) received more frequently an effective and appropriate antibiotic therapy than patients receiving conventional strategy on the first day of BSI diagnosis. Studies in settings where the prevalence of Ambler class A producers is high must be done to definitively conclude on the interest of the rapid technique.

In conclusion, the two evaluated assays (BL-RED^TM^ and β-LACTA^TM^ tests) showed excellent PPV, at least for the detection of the common Ambler class A family conferring 3GC-resistance and are suitable for the routine microbiology laboratory, decreasing the time to detection, and the need to refer these isolates to molecular biology confirmation. When a positive result is detected, these techniques permitted a quick and easy detection of the presence of ESBL-producing Enterobacterales in a clinical sample. They may help clinicians to guide appropriate antimicrobial therapy in septic patients and presumably improve their prognosis in a country or region where the Ambler class A family is particularly prevalent.

## Data Availability Statement

All datasets presented in this study are included in the article/Supplementary Material.

## Author Contributions

CD has performed experiments, statistical analysis, and wrote the manuscript. AB has performed experiments and critically reviewed the manuscript. J-PL and AP have conceived the study, discussed the results, and wrote the manuscript. All authors contributed to the article and approved the submitted version.

## Conflict of Interest

The authors declare that the research was conducted in the absence of any commercial or financial relationships that could be construed as a potential conflict of interest.
