# Supplementary material for: Evaluation of Two Methods for the Detection of Third Generation Cephalosporins Resistant Enterobacterales Directly From Positive Blood Cultures
Source: Front Cell Infect Microbiol. 2020 Sep 11;10:491. doi: 10.3389/fcimb.2020.00491 (PMC7516202; doi:10.3389/fcimb.2020.00491)
Supplement: Supplementary file 1 [file Table_1.DOCX]

Supplementary Material

# Supplementary Tables

| **Table S1. Performance of the BL-RED^TM^ and β-LACTA^TM^ tests on Class A ß-lactamases-producing isolates and main results of disc-diffusion method.** | | | | | | | | |
| --- | --- | --- | --- | --- | --- | --- | --- | --- |
| **Group (no. of strains)** | **Species (no. of strains)** | **β-Lactamases (no. of strains)** | **BL RED^TM^ results (nA)** | **β -LACTA^TM^ test results** | **Diameters of Ceftazidime 10µg disc (mm)^†^** | **Diameters of Cefotaxime 5µg disc (mm)^†^** | **Diameters of Ertapenem 10µg disc (mm)^†^** | |
| **ESBL (29)*** | *E. coli* (16) | CTX-M-group 1 (7) | Positive (680) | Positive | 21 | 6 | 34 | |
|  |  |  | Positive (770) | Positive | 17 | 6 | 34 | |
|  |  |  | Positive (840) | Positive | 16 | 6 | 29 | |
|  |  |  | Positive (350) | Positive | 13 | 6 | 34 | |
|  |  |  | Positive (760) | Positive | 11 | 6 | 33 | |
|  |  |  | Positive (659.99) | Positive | 6 | 6 | 27 | |
|  |  |  | Positive (849.99) | Positive | 6 | 6 | 28 | |
|  |  | CTX-M-group 1 + CTX-M-group 8 (5) | Negative (0) | Positive | 14 | 6 | 31 | |
|  |  |  | Positive (429.99) | Positive | 16 | 6 | 33 | |
|  |  |  | Positive (590) | Positive | 18 | 6 | 32 | |
|  |  |  | Positive (210) | Positive | 21 | 6 | 30 | |
|  |  |  | Positive (699.99) | Positive | 18 | 6 | 36 | |
|  |  | CTX-M-group 9 (4) | Positive (100) | Positive | 27 | 11 | 37 | |
|  |  |  | Negative (0) | Positive | 17 | 6 | 34 | |
|  |  |  | Positive (820) | Positive | 19 | 6 | 36 | |
|  |  |  | Positive (1000) | Positive | 23 | 6 | 33 | |
|  |  |  |  |  |  |  |  | |
|  | *P. mirabilis* (5) | CTX-M-group 1 (3) | Negative (0) | Positive | 24 | 16 | 34 | |
|  |  |  | Positive (690) | Positive | 30 | 16 | 38 | |
|  |  |  | Negative (0) | Positive | 24 | 6 | 32 | |
|  |  | CTX-M no group typed (1) | Positive (930) | Positive | 28 | 6 | 35 | |
|  |  | CTX-M-group 9 (1) | Positive (940) | Positive | 30 | 6 | 34 | |
|  |  |  |  |  |  |  |  | |
|  | *K. pneumoniae* (4) | CTX-M-group 1 (2) | Positive (580) | Positive | 16 | 6 | 33 | |
|  |  |  | Positive (820) | Positive | 6 | 6 | 10 | |
|  |  | CTX-M-group 1+ CTX-M-group 8 (1) | Positive (979.99) | Positive | 11 | 6 | 29 | |
|  |  | SHV-5 (1) | Negative (10) | Positive | 6 | 6 | 21 | |
|  |  |  |  |  |  |  |  | |
|  | *E. cloacae* (3) | CTX-M-group 1 + Hyperproduced AmpC (1) | Positive (1220) | Positive | 6 | 6 | 25 | |
|  |  | CTX-M-group 9 + Hyperproduced AmpC (1) | Negative (10) | Positive | 6 | 6 | 16** | |
|  |  | TEM-24 + Hyperproduced AmpC (1) | Positive (440) | Positive | 6 | 6 | 16** | |
|  |  |  |  |  |  |  |  | |
|  | *K. oxytoca* (1) | CTX-M-group 8 (1) | Positive (390) | Positive | 16 | 17 | 31 | |
| **OXY (3)** | *K. oxytoca* (3) | Hyperproduced OXY (3) | Positive (1100) | Positive | 17 | 6 | 21** | |
|  |  |  | Positive (810) | Positive | 26 | 8 | 23** | |
|  |  |  | Positive (1069.99) | Positive | 6 | 6 | 16** | |
| **KPC (5)** | *K. pneumoniae* (5) | KPC-2 (4) | Positive (810) | Positive | 26 | 8 | 23 | |
|  |  |  | Positive (1069.99) | Positive | 6 | 6 | 16 | |
|  |  |  | Positive (410) | Positive | 8 | 6 | 6 | |
|  |  |  | Positive (710) | Positive | 6 | 6 | 6 | |
|  |  | KPC-2 + CTX-M-group 1 (1) | Positive (900) | Positive | 16 | 13 | 15 | |
| **IMI (1)** | *E. cloacae* (1) | IMI-1 (1) | Negative (0) | Negative | 31 | 33 | 13 | |
| * Excluding isolates co-producing carbapenemase  ** Isolates associating membrane permeability alteration  **^†^** Susceptibility patterns were interpreted according to EUCAST/SFM 2019 guidelines: Isolates were resistant if critical diameters were <19 mm for ceftazidime,<17 mm for cefotaxime and <22 mm for ertapenem | | | | | | | |  |

| **Table S2. Performance of the BL-RED^TM^ and β-LACTA^TM^ tests on Class C ß-lactamases-producing isolates and main results of disc-diffusion method.** | | | | | | | | | | | | |  |
| --- | --- | --- | --- | --- | --- | --- | --- | --- | --- | --- | --- | --- | --- |
| **Group (no. of strains)** | **Species (no. of strains)** | **β-Lactamases (no. of strains)** | **BL RED^TM^ results (nA)** | | **β-LACTA^TM^ test results** | | **Diameters of Ceftazidime 10µg disc (mm)^†^** | | **Diameters of Cefotaxime 5µg disc (mm)^†^** | | **Diameters of Ertapenem**  **10µg disc (mm)^†^** | |  |
| **Chromosomic AmpC (29)*** | *E. cloacae* (9) | Hyperproduced AmpC (9) | Negative (0) | | Negative | | 6 | | 6 | | 25 | |  |
|  |  |  | Negative (0) | | Negative | | 6 | | 10 | | 26 | |  |
|  |  |  | Negative (0) | | Negative | | 6 | | 6 | | 25 | |  |
|  |  |  | Negative (0) | | Negative | | 6 | | 6 | | 14** | |  |
|  |  |  | Negative (20) | | Negative | | 6 | | 6 | | 21** | |  |
|  |  |  | Negative (0) | | Negative | | 6 | | 6 | | 18** | |  |
|  |  |  | Negative (0) | | Negative | | 6 | | 6 | | 17** | |  |
|  |  |  | Negative (0) | | Negative | | 6 | | 6 | | 22** | |  |
|  |  |  | Negative (0) | | Negative | | 6 | | 6 | | 22** | |  |
|  |  |  |  | |  | |  | |  | |  | |  |
|  | *K. aerogenes* (6) | Hyperproduced AmpC (6) | Negative (0) | | Negative | | 9 | | 8 | | 28 | |  |
|  |  |  | Negative (0) | | Negative | | 9 | | 6 | | 27 | |  |
|  |  |  | Negative (0) | | Negative | | 8 | | 8 | | 28 | |  |
|  |  |  | Negative (0) | | Negative | | 6 | | 6 | | 25 | |  |
|  |  |  | Negative (0) | | Negative | | 8 | | 6 | | 25 | |  |
|  |  |  | Negative (0) | | Negative | | 13 | | 11 | | 25 | |  |
|  |  |  |  | |  | |  | |  | |  | |  |
|  | *E. coli* (6) | Hyperproduced AmpC (6) | Negative (0) | | Negative | | 9 | | 11 | | 32 | |  |
|  |  |  | Negative (0) | | Negative | | 6 | | 6 | | 27 | |  |
|  |  |  | Negative (0) | | Negative | | 20 | | 19 | | 32 | |  |
|  |  |  | Negative (0) | | Negative | | 22 | | 16 | | 35 | |  |
|  |  |  | Negative (0) | | Negative | | 19 | | 15 | | 30 | |  |
|  |  |  | Negative (0) | | Negative | | 19 | | 18 | | 33 | |  |
|  |  |  | |  | |  | |  | |  | |  | |
|  | *C. freundii* (4) | Hyperproduced AmpC (4) | Negative (0) | | Negative | | 6 | | 6 | | 28 | |  |
|  |  |  | Negative (60) | | Negative | | 6 | | 6 | | 30 | |  |
|  |  |  | Negative (0) | | Negative | | 6 | | 6 | | 29 | |  |
|  |  |  | Negative (0) | | Negative | | 6 | | 6 | | 16** | |  |
|  |  |  |  | |  | |  | |  | |  | |  |
|  | *M. morganii* (3) | Hyperproduced AmpC (3) | Negative (0) | | Negative | | 11 | | 8 | | 33 | |  |
|  |  |  | Negative (0) | | Negative | | 15 | | 13 | | 34 | |  |
|  |  |  | Negative (0) | | Negative | | 12 | | 9 | | 34 | |  |
|  |  |  |  | |  | |  | |  | |  | |  |
|  | *H. alvei* (1) | Hyperproduced AmpC (1) | Negative (0) | | Negative | | 18 | | 22 | | 36 | |  |
|  |  |  |  | |  | |  | |  | |  | |  |
| **Plasmidic AmpC (9)*** | *K. pneumoniae* (9) | DHA-1 (9) | Negative (0) | | Negative | | 6 | | 6 | | 27 | |  |
|  |  |  | Negative (0) | | Negative | | 6 | | 6 | | 19** | |  |
|  |  |  | Positive (480) | | Negative | | 6 | | 6 | | 14** | |  |
|  |  |  | Negative (0) | | Negative | | 14 | | 6 | | 18** | |  |
|  |  |  | Negative (0) | | Negative | | 6 | | 6 | | 18** | |  |
|  |  |  | Negative (0) | | Negative | | 6 | | 6 | | 15** | |  |
|  |  |  | Negative (0) | | Negative | | 9 | | 6 | | 22** | |  |
|  |  |  | Negative (0) | | Negative | | 6 | | 6 | | 23** | |  |
|  |  |  | Negative (0) | | Negative | | 6 | | 6 | | 14** | |  |
| * Excluding isolates co-producing ESBL and/or carbapenemase  ** Isolates associating membrane permeability alteration  **^†^** Susceptibility patterns were interpreted according to EUCAST/SFM 2019 guidelines: Isolates were resistant if critical diameters were <19 mm for ceftazidime,<17 mm for cefotaxime and <22 mm for ertapenem | | | | | | | | | | | | |  |

| **Table S3. Performance of the BL-RED^TM^ and β-LACTA^TM^ tests on Class B and Class D ß-lactamases-producing isolates and main results of disc-diffusion method.** | | | | | | | | |
| --- | --- | --- | --- | --- | --- | --- | --- | --- |
| **Group (no. of strains)** | **Carbapenemase type (no. of strains)** | **Species (no. of strains)** | **β-Lactamases (no. of strains)** | **BL RED^TM^ results (nA)** | **β-LACTA^TM^ test results** | **Diameters of Ceftazidime 10µg disc (mm)^†^** | **Diameters of Cefotaxime 5µg disc (mm)^†^** | **Diameters of Ertapenem 10µg disc (mm)^†^** |
| **Class B (10)** | NDM (5) | *K. pneumoniae* (2) | NDM-1 + CTX-M-group 1 (2) | Positive (909.99) | Positive | 6 | 6 | 6 |
|  |  |  |  | Positive (870) | Positive | 6 | 6 | 13 |
|  |  |  |  |  |  |  |  |  |
|  |  | *E. coli* (2) | NDM-1 + CTX-M-group 9 (1) | Positive (359.99) | Positive | 11 | 6 | 20 |
|  |  |  | NDM-1 + DHA (1) | Negative (0) | Negative | 6 | 6 | 18 |
|  |  |  |  |  |  |  |  |  |
|  |  | *P. mirabilis* (1) | NDM-1 (1) | Negative (0) | Negative | 6 | 6 | 27 |
|  |  |  |  |  |  |  |  |  |
|  | VIM (5) | *E. cloacae* (4) | VIM + Hyperproduced AmpC (3) | Negative (0) | Negative | 14 | 6 | 14 |
|  |  |  |  | Negative (0) | Negative | 6 | 6 | 15 |
|  |  |  |  | Negative (0) | Negative | 6 | 6 | 15 |
|  |  |  | VIM + CTX-M-group 9 (1) | Positive (269.99) | Positive | 6 | 6 | 18 |
|  |  |  |  |  |  |  |  |  |
|  |  | *C. freundii* (1) | VIM + Hyperproduced AmpC (1) | Negative (0) | Negative | 6 | 6 | 31 |
|  |  |  |  |  |  |  |  |  |
| **Class D (12)** | OXA-48 (11) | *K. pneumoniae* (4) | OXA-48 (2) | Negative (0) | Negative | 27 | 13 | 6 |
|  |  |  |  | Negative (0) | Negative | 27 | 20 | 18 |
|  |  |  | OXA-48 + CTX-M-group 1 (2) | Positive (960) | Positive | 6 | 6 | 20 |
|  |  |  |  | Positive (880) | Positive | 6 | 6 | 20 |
|  |  |  |  |  |  |  |  |  |
|  |  | *E. cloacae* (2) | OXA-48 + CTX-M-group 1 (2) | Positive (900) | Positive | 6 | 6 | 17 |
|  |  |  |  | Positive (950) | Positive | 10 | 6 | 14 |
|  |  |  |  |  |  |  |  |  |
|  |  | *K. aerogenes* (2) | OXA-48 (2) | Negative (0) | Negative | 25 | 20 | 18 |
|  |  |  |  | Negative (0) | Negative | 26 | 24 | 23 |
|  |  |  |  |  |  |  |  |  |
|  |  | *K. oxytoca* (1) | OXA-48 (1) | Negative (0) | Negative | 31 | 20 | 21 |
|  |  |  |  |  |  |  |  |  |
|  |  | *E. coli* (1) | OXA-48 (1) | Negative (0) | Negative | 30 | 25 | 24 |
|  |  |  |  |  |  |  |  |  |
|  |  | *S. marcescens* (1) | OXA-48 (1) | Negative (0) | Negative | 28 | 16 | 20 |
|  |  |  |  |  |  |  |  |  |
|  | OXA-181 (1) | *E. coli* (1) | OXA-181 + CTX-M-group 1 (1) | Positive (680) | Positive | 6 | 6 | 18 |
|  |  |  |  |  |  |  |  |  |
| **Class B + D (2)** | NDM + OXA-48 (2) | *K. pneumoniae* (2) | NDM-1 + OXA-48 + CTX-M-group 1 (2) | Positive (860) | Positive | 6 | 6 | 6 |
|  |  |  |  | Positive (800) | Positive | 6 | 6 | 13 |

## ^†^ Susceptibility patterns were interpreted according to EUCAST/SFM 2019 guidelines: Isolates were resistant if critical diameters were <19 mm for ceftazidime,<17 mm for cefotaxime and <22 mm for ertape
